# CRISPR/Cas13d-Mediated Microbial RNA Knockdown

**DOI:** 10.3389/fbioe.2020.00856

**Published:** 2020-07-30

**Authors:** Kun Zhang, Zhihui Zhang, Jianan Kang, Jiuzhou Chen, Jiao Liu, Ning Gao, Liwen Fan, Ping Zheng, Yu Wang, Jibin Sun

**Affiliations:** ^1^Key Laboratory of Systems Microbial Biotechnology, Tianjin Institute of Industrial Biotechnology, Chinese Academy of Sciences, Tianjin, China; ^2^University of Chinese Academy of Sciences, Beijing, China; ^3^College of Life Engineering, Shenyang Institute of Technology, Fushun, China; ^4^School of Life Sciences, University of Science and Technology of China, Hefei, China

**Keywords:** RNA knockdown, CRISPR, Cas13d, CasRx, type IV-D CRISPR effector

## Abstract

RNA-guided and RNA-targeting type IV-D CRISPR/Cas systems (CRISPR/Cas13d) have recently been identified and employed for efficient and specific RNA knockdown in mammalian and plant cells. Cas13d possesses dual RNase activities and is capable of processing CRISPR arrays and cleaving target RNAs in a protospacer flanking sequence (PFS)-independent manner. These properties make this system a promising tool for multiplex gene expression regulation in microbes. Herein, we aimed to establish a CRISPR/Cas13d-mediated RNA knockdown platform for bacterial chassis. CasRx, Cas13d from *Ruminococcus flavefaciens* XPD3002, was selected due to its high activity. However, CasRx was found to be highly toxic to both *Escherichia coli* and *Corynebacterium glutamicum*, especially when it cooperated with its guide and target RNAs. After employing a low copy number vector, a tightly controlled promoter, and a weakened ribosome binding site, we successfully constructed an inducible expression system for CasRx and applied it for repressing the expression of a green fluorescent protein (GFP) in *E. coli*. Despite our efforts to optimize inducer usage, guide RNA (gRNA) architecture and combination, and target gene expression level, the highest gene repression efficiency was 30–50% at the protein level and ∼70% at the mRNA level. The moderate RNA knockdown is possibly caused by the collateral cleavage activity toward bystander RNAs, which acts as a mechanism of type IV-D immunity and perturbs microbial metabolism. Further studies on cellular response to CRISPR/Cas13d and improvement in RNA knockdown efficiency are required prior to practical application of this system in microbes.

## Introduction

Clustered regularly interspaced short palindromic repeat (CRISPR)/CRISPR-associated protein (Cas) systems that endow microbes with diverse mechanisms for adaptive immunity have been widely engineered to facilitate gene editing even in some genetically intractable microbes (Shapiro et al., 2018; Zheng et al., 2020). However, to facilitate rapid mapping of gene expression levels to metabolic outputs, targeted gene regulation techniques are also in demand. To this end, catalytically dead versions of RNA-guided and DNA-targeting type II (Cas9) and type V (Cas12) systems have been repurposed for CRISPR interference (CRISPRi) in microbes (Lian et al., 2017; Otoupal and Chatterjee, 2018; Li et al., 2020). Such techniques require a protospacer adjacent motif (PAM) for target recognition. Another concern of DNA-targeting CRISPRi is that targeting a gene in an operon will cause a collateral effect, which silences transcription of downstream genes (Cui et al., 2018). In addition to gene perturbation at transcription stage, RNA interference (RNAi) that can modulate gene expression at the translation stage has also been developed using synthetic RNAs (Na et al., 2013; Laganà et al., 2014). However, functioning of RNAi depends on proper host machinery and thus is limited to certain organisms. It is also suggested that RNAi sometimes exhibit significant off-target effects (Qi et al., 2013).

**FIGURE 1:**
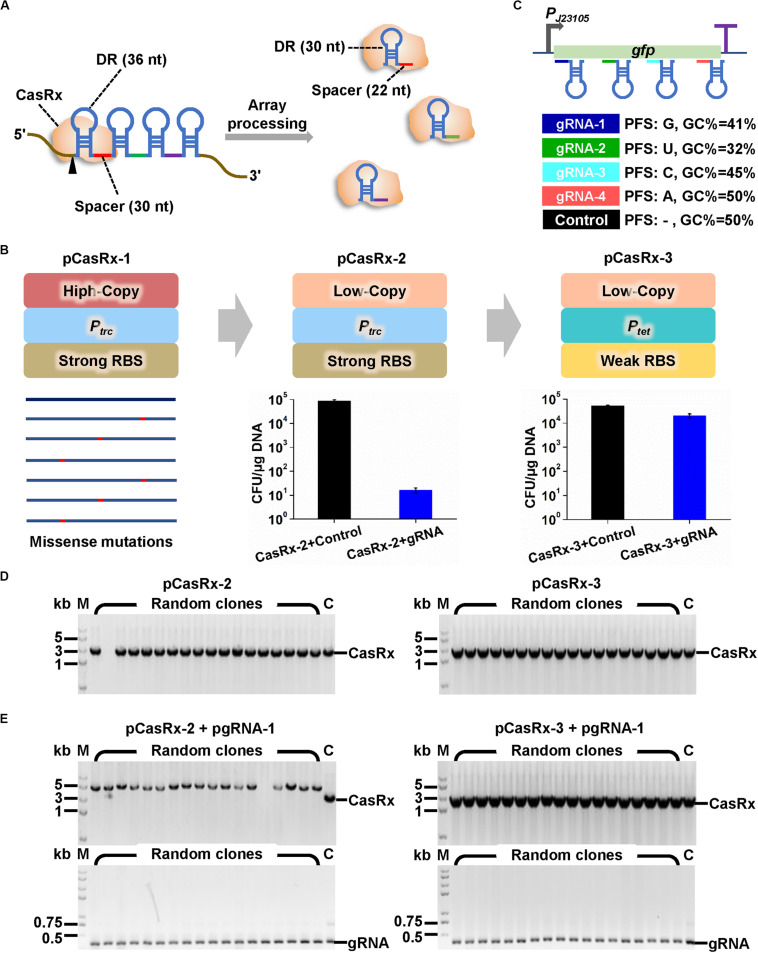
Construction of microbial expression systems for CRISPR/CasRx. **(A)** Multiple gRNAs containing a 30-nt spacer flanked by two 36-nt DRs can be expressed as a single array and processed by CasRx into individual mature gRNAs containing 30-nt DR and 22-nt spacer. **(B)** Strategies to construct *casRx* expression plasmid for *E. coli*. The high copy number *pUC* replicon was first replaced with a low copy number *pSC101* replicon. An IPTG inducible promoter *P*_*tac*_ and a strong RBS (AAAGGAGTTGAGA) were then replaced with a Tc inducible promoter *P*_*tet*_ and a weak RBS (AAAGGCACCCGAT). To determine plasmid transformation efficiency, *E. coli* harboring pGFP was co-transformed with a *casRx* expression plasmid (pCasRx-2 or pCasRx-3) and an empty plasmid with no gRNA cassette (control) or a *gfp*-targeting gRNA expression plasmid (pgRNA-1). Cells were plated on LB solid plates supplemented with Amp + Cm + Kan. Clones were counted after 24 h cultivation. **(C)** Design of four *gfp*-targeting pre-gRNAs with different characteristics and a non-targeting control with no target site on the whole *E. coli* and *C. glutamicum* transcripts. **(D)** Clone PCR verification of transformants of pCasRx-2 or pCasRx-3. M, DNA marker; C, plasmid positive control. **(E)** Clone PCR verification of transformants of pCasRx-2 + pgRNA-1 or pCasRx-3 + pgRNA-1. M, DNA marker; C, plasmid positive control.

Newly discovered type IV CRISPR systems are RNA-guided and RNA-targeting systems, which include a single protein effector (Cas13) that can target and cleave a specific RNA with a single guide RNA (gRNA) (Shmakov et al., 2015; Abudayyeh et al., 2016; Smargon et al., 2017). Cas13 is also capable to process a CRISPR repeat array into mature gRNAs via a HEPN domain-independent mechanism (Figure 1A). These properties facilitate rapid development of a new generation of RNA targeting and editing tools for multiplex gene regulation. Type IV systems can be divided into four subtypes (A–D) based on the phylogeny of effector complexes (Wang F. et al., 2019). Cas13a/b/c were initially discovered and their applications in RNA knockdown in mammalian cells exhibited high efficiency and specificity (Abudayyeh et al., 2017; Cox et al., 2017). Instead of a preferred PAM sequence, Cas13a requires a 3′ protospacer flanking sequence (PFS) of H, while Cas13b requires both a 3′ PFS of NAN or NNA and a 5′ PFS of D for effective RNA cleavage (Abudayyeh et al., 2016; Smargon et al., 2017). Type IV-D CRISPR effectors, known as Cas13d, were recently discovered and employed for RNA knockdown in mammalian cells. In contrast to other RNA-targeting systems, target RNA cleavage by CRISPR/Cas13d is PFS-independent (Konermann et al., 2018; Yan et al., 2018; Zhang C. et al., 2018). Notably, Cas13d from *Ruminococcus flavefaciens* XPD3002 (CasRx) was reported to mediate more efficient and specific RNA knockdown in plants than frequently used Cas13a and Cas13b variants (Mahas et al., 2019). Although *Escherichia coli* was used as a bacterial host for functional screening for CRISPR/Cas13d (Yan et al., 2018), this system has not been characterized in detail in microbes and employed as a microbial gene regulation tool yet.

In this study, we aimed to develop a microbial RNA knockdown technique based on the most promising CRISPR/Cas13d system, CRISPR/CasRx. Unexpectedly, we found that CRISPR/CasRx system was highly toxic to two important platform microbes *E. coli* and *Corynebacterium glutamicum*. After lowering the plasmid copy number and weakening the leaky expression of the *casRx* gene, a plasmid-based expression system for CRISPR/CasRx was constructed. Using green fluorescent protein (GFP) as a reporter, moderate gene repression was achieved in *E. coli* with this system, which was verified at both mRNA and protein levels. Various RNA architectures and combinations were also tested for their effects on RNA knockdown efficiency.

## Materials and Methods

### Bacterial Strains and Growth Conditions

Bacterial strains used in this study are listed in Supplementary Table S1. *E. coli* Trans1-T1 (Transgen, China) was used as the host strain for general cloning and RNA knockdown test. *E. coli* DB 3.1 was used for cloning plasmids harboring the *ccdB* gene. *E. coli* strains were cultivated in a Luria–Bertani (LB) medium at 37°C and with shaking at 220 rpm. Ampicillin (Amp, 100 μg/mL), chloramphenicol (Cm, 20 μg/mL), or kanamycin (Kan, 50 μg/mL) was added when only one antibiotic was used. In the case in which multiple antibiotics were added to maintain multiple plasmids, antibiotics were used at lower concentrations (Amp, 75 μg/mL; Cm, 10 μg/mL; Kan, 25 μg/mL). Tetracycline (Tc) was added at the beginning of cultivation to induce *casRx* expression. A *C. glutamicum* ATCC 13032 derivative harboring a chromosomal *gfp* expression cassette was used to test the transformation of *casRx* expression plasmid and cultivated aerobically at 30°C in an LB medium supplemented with 5 g/L glucose. Electro-competent cells were prepared as described previously (Ruan et al., 2015).

### Plasmid Construction

Plasmids used in this study are listed in Supplementary Table S1. The *casRx* gene was synthesized by GenScript (China) and first cloned to an *E. coli*–*C. glutamicum* shuttle plasmid pXMJ19 under the control of isopropyl-β-D-thiogalactopyranoside (IPTG) inducible promoter *P*_*tac*_, producing plasmid pCasRx-1. To correct the unexpected mutations in *casRx* and lower the copy number of pCasRx-1, two fragments were amplified from pCasRx-1 with primer pairs CasRx-F1/CasRx-R1 and CasRx-F2/CasRx-R2 to correct the mutations and remove the original *pUC* replicon. *pSC101* replicon was amplified from plasmid pSB4K5-I52002 (Shetty et al., 2008) with a primer pair pSC101-F/pSC101-R and ligated with the aforementioned two fragments using a ClonExpress MultiS One Step Cloning Kit (Vazyme, China), producing pCasRx-2. To achieve Tc inducible expression of *casRx* and change the original strong RBS (AAAGGAGTTGAGA) to a weaker one (AAAGGCACCCGAT), *casRx* was amplified from pCasRx-2 with a primer pair CasRx-F3/CasRx-R3. A weaker RBS was simultaneously added with the forward primer. A laboratory stock plasmid pZSA harboring a *P*_*tet*_ promoter and a TetR effector encoding gene was linearized by PCR with a primer pair pZSA-F/pZSA-R. The *casRx* and pZSA fragments were ligated to produce pCasRx-3.

An *E. coli*–*C. glutamicum* shuttle plasmid pgRNA-*ccdB* (pEC-XK99E backbone) (Wang et al., 2018b) for expressing gRNA of Cas9 was used as a template to construct plasmids pgRNA-*ccdB*-1 and pgRNA-*ccdB*-2, which were designed to express unprocessed pre-gRNA [a 30-nt spacer flanked by two 36-nt direct repeats (DRs)] and mature gRNA (a 30-nt DR with a 22-nt spacer) of CasRx, respectively (Supplementary Figure S1). gRNA transcription was controlled by a constitutive promoter *P_11__*F*_* that functions in both *E. coli* and *C. glutamicum* (Liu et al., 2017). The backbone of pgRNA-*ccdB* was amplified with a primer pair pgRNA-F/pgRNA-R. *ccdB* fragment was amplified and DRs were simultaneously added with primer pairs DR36-*ccdB*-F/DR36-*ccdB*-R (or DR30-*ccdB*-F/DR30-*ccdB*-R). The plasmid backbone and *ccdB* fragment were ligated to produce pgRNA-*ccdB*-1 (or pgRNA-*ccdB*-2). To construct an expression plasmid for *gfp*-targeting gRNA, a primer pair was used to generate double-stranded DNA (dsDNA) containing a *gfp*-targeting spacer. The dsDNA was assembled with pgRNA-*ccdB*-1 (or pgRNA-*ccdB*-2) to replace *ccdB* with the spacer by Golden Gate assembly described previously (Wang et al., 2018b; Supplementary Figure S1). To insert additional nucleotides between the second 36-nt DR and a terminator, an *ldhA* fragment was amplified from *E. coli* genomic DNA with primer pair adn-F/adn-100-R, adn-F/adn-300-R, or adn-F/adn-1000-R. The *ldhA* fragment and a dsDNA containing spacer and 36-nt DR were assembled with pgRNA-*ccdB*-1 by Golden Gate assembly.

To construct a GFP reporter system that is compatible with the CRISPR/CasRx system, pGFP was constructed with the pTrc99A backbone, the *p15A* replicon, and the *gfp* gene. *gfp* was first amplified from pTRCmob-*egfp* (Wang et al., 2018a) with a primer pair GFP-F/GFP-R, and a constitutive promoter *P_*J*__23105_* was added to the PCR product via the forward primer. Two fragments were amplified from pTrc99A by PCR with primer pairs pTrc99A-F1/pTrc99A-R1 and pTrc99A-F2/pTrc99A-R2 to remove the original *pUC* replicon. p15A replicon was amplified from pACYCDuet-1 by PCR with a primer pair p15A-F/p15A-R. The three PCR products were ligated to produce pGFP. To construct a reporter system with a lower GFP expression level, pGFP-w was constructed by PCR using pGFP as a template and the primer pair J23117-F/J23117-R. The promoter *P_*J*__23105_* was replaced with a weaker promoter *P_*J*__23117_*. Primers used for plasmid construction, spacers, and additional nucleotides in gRNAs are listed in Supplementary Tables S2–S4, respectively. The full sequences for all the plasmids constructed in this study were uploaded as Supplementary Material in GenBank format.

### Assay of RNA Knockdown Efficiency by Determining GFP Fluorescence

*E. coli* harboring pGFP or pGFP-w was co-transformed with pCasRx-3 and a gRNA expression plasmid. Cells were plated in LB solid plates containing Amp, Cm, and Kan. After 24-h cultivation at 37°C, clones were verified by PCR, and three correct clones were picked and incubated in a 5 mL LB medium supplemented with antibiotics. The cultures were used as seeds to inoculate a 10 mL fresh LB medium in 100 mL shake flasks supplemented with antibiotics and inducer. The initial cell density (OD_600__*nm*_) was set as 0.05. During cultivation, cells were collected periodically by centrifugation at 6,000 × *g* for 5 min, washed once with phosphate buffered saline (PBS) buffer (pH 7.4), and resuspended in PBS buffer in Corning 3603 96-well microplates (Corning Incorporated, United States). GFP fluorescence was measured using an Infinite 200 PRO plate reader (Tecan Trading AG, Switzerland) (λ excitation = 488 nm, λ emission = 520 nm), and OD_600__*nm*_ was determined simultaneously.

### Transcription Level Assay by Real-Time Quantitative PCR (RT-qPCR)

Total RNAs were extracted from cells of exponential phase using an RNAprep Pure Cell/Bacteria Kit, treated with DNase I, and used to synthesize cDNAs using random primers and a Fast Quant RT Kit. The resultant cDNAs were used as templates for RT-qPCR analysis. The total RNA samples were also used as templates for RT-qPCR to confirm that genomic DNA contamination during total RNA extraction was minimal. Specific primers for RT-qPCR were designed using Beacon Designer software v7.7 (PREMIER Biosoft International, United States) (Supplementary Table S2). RT-qPCR was performed using a SuperReal Premix SYBR Green Kit and Applied Biosystems^®^ 7500 Real-Time PCR System (Thermo Fisher Scientific, United States) according to the manufacturers’ instructions. The relative transcription level of *gfp* was calculated using the 2^–Δ^ ^Δ^ ^*CT*^ method. All the kits used for RT-qPCR were purchased from Tiangen Biotech, China.

## Results

### Construction of Bacterial Expression Systems for CasRx and gRNA

Development of a gene regulation technique based on CRISPR/Cas13d requires functional and controllable expression of a Cas13d effector and transcription of a gRNA with proper architecture. CRISPR/CasRx system (Konermann et al., 2018) that has been successfully applied in RNA knockdown in mammalian and plant cells was used here. We intended to develop a versatile CRISPR/CasRx-mediated RNA knockdown platform for two widely used microbial chassis, *E. coli* and *C. glutamicum*. Therefore, *casRx* was synthesized and cloned to pXMJ19, an *E. coli*–*C. glutamicum* shuttle vector with a high copy number *E. coli* replicon (*pUC*, ∼75 copies/cell) and a low copy number *C. glutamicum* replicon (*pBL1*, ∼8 copies/cell) (Jakoby et al., 1999). In the recombinant plasmid pCasRx-1, *casRx* was controlled by an IPTG inducible promoter *P*_*tac*_. However, few transformants of ligation products were obtained, and gene sequencing suggested existence of missense mutations in *casRx* in all sequenced plasmids (Figure 1B). Considering the possible leaky expression of *P*_*tac*_ and the cellular toxicity of Cas effectors to microbes (Liu et al., 2017; Wang K. et al., 2019), the high copy number *pUC* replicon was replaced by *pSC101* replicon with a low copy number in *E. coli* (∼8 copies/cell). This modification produced recombinant plasmid pCasRx-2 harboring the correct *casRx* gene.

Next, gRNA expression plasmids were constructed based on another *E. coli*–*C. glutamicum* shuttle vector pEC-XK99E with a high copy number *E. coli* replicon (*pUC*, ∼75 copies/cell) and a medium copy number *C. glutamicum* replicon (*pGA1*, ∼30 copies/cell) (Kirchner and Tauch, 2003). The gRNA was initially designed as a 30-nt spacer flanked by two 36-nt DRs to mimic an unprocessed pre-gRNA. The secondary structure of GFP reporter gene transcript was predicted by using the Vienna RNA websuite^1^ (Gruber et al., 2008; Supplementary Figure S2). Four gRNAs whose spacers target distinct regions of *gfp* transcript and possess different G + C contents and PFSs were designed (Figure 1C and Supplementary Figure S2). A non-targeting gRNA with no target site on the whole transcripts of *E. coli* and *C. glutamicum* was designed as a control. Then, plasmid pCasRx-2 was transformed into *E. coli* harboring pGFP together with plasmid pgRNA-1 or an empty plasmid with no gRNA cassette. Unexpectedly, co-transformation of pCasRx-2 and pgRNA-1 significantly reduced the number of transformants by approximately 1,000-fold, compared to the transformation of pCasRx-2 and empty plasmid (Figure 1B). Clone PCR verification suggested that when pCasRx-2 and pgRNA-1 were co-transformed, the resultant transformants possessed incorrect *casRx* gene (Figures 1D,E).

According to previous literatures, when the Cas13d effector combines with gRNA and target mRNA to form a ternary Cas13d-gRNA-target mRNA complex, the complex is capable of cleaving non-specific bystander RNAs (Konermann et al., 2018; Yan et al., 2018). Such bystander cleavage may disturb cell metabolism or even be lethal to cells. We speculated that a tighter regulation of *casRx* transcription and a weaker translation initiation may be essential for successful development of a functional CRISPR/CasRx system in microbes. Therefore, the IPTG inducible *P*_*tac*_ promoter and original strong RBS (AAAGGAGTTGAGA) in pXMJ19 were replaced with a Tc inducible *P*_*tet*_ promoter with better tightness (Lutz and Bujard, 1997) and a weaker RBS (AAAGGCACCCGAT). This modification significantly increased co-transformation efficiency of the resultant plasmid pCasRx-3 and pgRNA-1 (Figure 1B). Clone PCR verification suggested that the transformants of pCasRx-3 and pgRNA-1 all harbored correct *casRx* gene (Figures 1D,E).

With efforts to recruit a low copy number replicon, a tightly controlled promoter, and a weak RBS, a CRISPR/CasRx expression system was established in *E. coli*. The pCasRx-2 plasmid that only has ∼8 copies per *C. glutamicum* cell was also tested for its maintenance in a *gfp* expressing *C. glutamicum*. However, non-transformants were obtained even though pCasRx-2 was transformed individually, suggesting that *C. glutamicum* might be more sensitive to CasRx than *E. coli* since the ternary Cas13d-gRNA-target mRNA complex was not generated due to the non-involvement of a gRNA expression plasmid.

### CRISPR/CasRx-Mediated RNA Knockdown in *E. coli*

**FIGURE 2:**
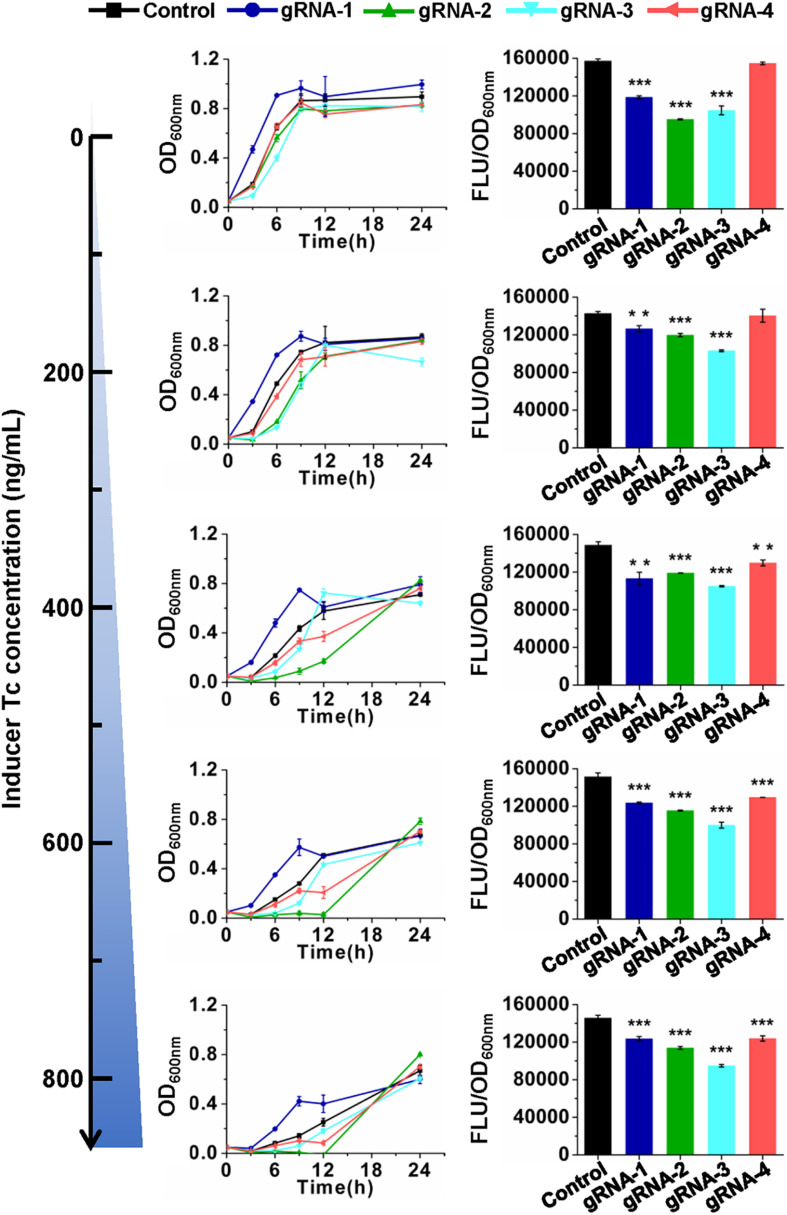
CRISPR/CasRx-mediated gene repression with pre-gRNAs. Design of gRNA-1, gRNA-2, gRNA-3, and gRNA-4 are shown in Figure 1B. The spacer sequences are listed in Supplementary Table S3. pCasRx-3 and different gRNA expression plasmids were co-transformed into *E. coli* harboring pGFP for gene repression. Inducer was added at the beginning of cultivation to induce *casRx* expression. GFP fluorescence and OD_600__*nm*_ were determined after 24-h cultivation. Error bars indicate standard deviations from three parallel experiments. All *t*-tests compare the GFP fluorescence per OD_600__*nm*_ using *gfp*-targeting gRNAs against non-targeting gRNA control (**P < 0.01, ***P < 0.001).

The developed CRISPR/CasRx system was then tested for its application in RNA knockdown using GFP as a reporter. pCasRx-3 was transformed into *E. coli* harboring pGFP with a gRNA plasmid harboring the *gfp*-targeting gRNA or non-targeting control gRNA, respectively (Figure 1C). Transformants were cultured in media containing different concentrations of Tc to explore the optimal inducer concentration for CRISPR/CasRx-mediated RNA knockdown. In the absence of an inducer, lowered GFP fluorescence was already detected for *gfp*-targeting gRNAs, except for gRNA-4 targeting the end of the *gfp* transcript (Figure 2). The result suggested that leaky expression of *casRx* still happened. With the increase in the Tc usage, growth inhibition and prolonged lag phase were observed, which may be caused by a reported Cas13-induced cellular dormancy (Meeske et al., 2019). However, the *gfp* repression efficiency was not significantly improved. Overall, the *gfp* repression efficiency fluctuated between 11.4 and 23.8%, 16.1 and 23.7%, and 27.7 and 34.8% for gRNA-1, gRNA-2, and gRNA-3, respectively. Up to 14.9% repression of *gfp* expression was observed for gRNA-4 (Figure 2).

**FIGURE 3:**
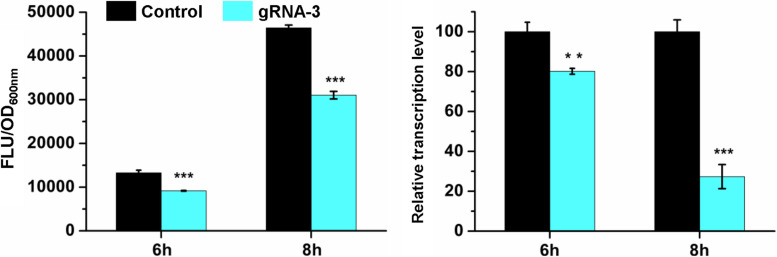
CRISPR/CasRx-mediated mRNA knockdown. pCasRx-3 and pgRNA-3 were co-transformed into *E. coli* harboring pGFP for gene repression. Inducer (200 ng/mL Tc) was added at the beginning of cultivation to induce *casRx* expression. Cells were collected at 6 and 8 h for GFP fluorescence and mRNA determination. Error bars indicate standard deviations from three parallel experiments. All *t*-tests compare the GFP fluorescence per OD_600__*nm*_ or *gfp* transcription level using *gfp*-targeting gRNA-3 against non-targeting gRNA control (**P < 0.01, ***P < 0.001).

To investigate whether the repression was caused by CRISPR/CasRx-mediated mRNA knockdown, RT-qPCR was conducted for the *gfp* repression test using gRNA-3 and CasRx induced by 200 ng/mL Tc due to relatively high repression efficiency and slight growth inhibition. Cells at the exponential phase were collected and analyzed. At 6 h when *gfp* was expressed at a relatively low level, *gfp* was repressed by 30.8% at the protein level and 19.8% at the mRNA level. At 8 h when *gfp* was expressed at a relatively high level, the repression efficiency increased to 33.1%, and the corresponding mRNA knockdown efficiency increased to 72.6% (Figure 3). The results suggest that the developed CRISPR/CasRx system can repress the expression of a target gene via knocking down the target mRNA in *E. coli*.

### Further Attempts to Improve RNA Knockdown Efficiency

**FIGURE 4:**
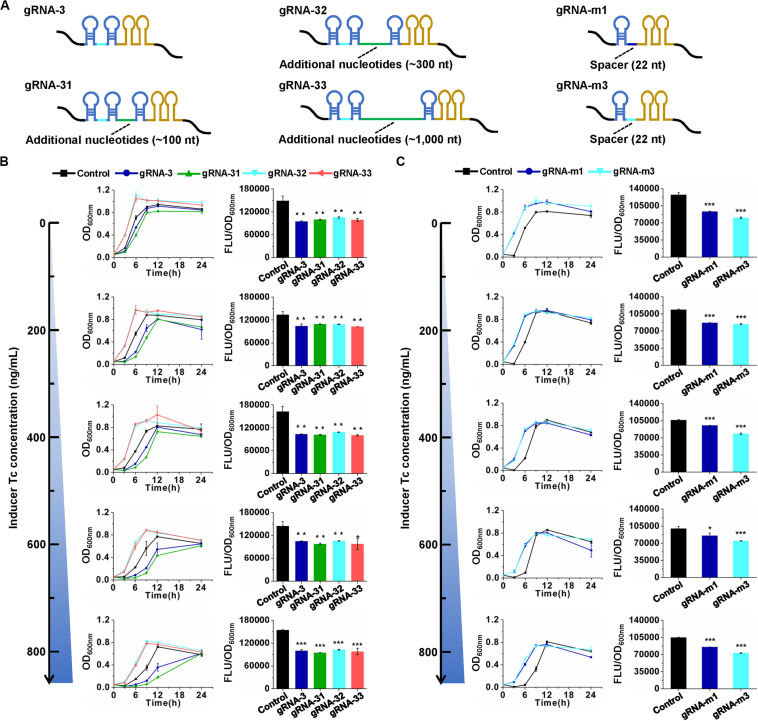
Effects of gRNA architecture on gene repression efficiency. **(A)** Architectures of pre-gRNAs containing 30-nt spacers and additional nucleotides and mature gRNAs containing 22-nt spacers. gRNA-31, gRNA-32, and gRNA-33 have the same spacer with gRNA-3 but additional nucleotides downstream of the second DR. gRNA-m1 and gRNA-m3 have 22-nt spacers, which are the same with the CasRx-processed gRNA-1 and gRNA-3, respectively. The spacer sequences are listed in Supplementary Table S3. **(B)** Growth and GFP fluorescence with pre-gRNAs and inducer at different concentrations. **(C)** Growth and GFP fluorescence with mature gRNAs and inducer at different concentrations. pCasRx-3 and different gRNA expression plasmids were co-transformed into *E. coli* harboring pGFP for gene repression. Inducer was added at the beginning of cultivation to induce *casRx* expression. GFP fluorescence and OD_600__*nm*_ were determined after 24 h cultivation. Error bars indicate standard deviations from three parallel experiments. All *t*-tests compare the GFP fluorescence per OD_600__*nm*_ using *gfp*-targeting gRNAs against non-targeting gRNA control (*P < 0.05, **P < 0.01, ***P < 0.001).

The knockdown effects of the CRISPR/CasRx system in *E. coli* are much lower compared to those in mammalian and plant cells (over 80%) (Konermann et al., 2018; Mahas et al., 2019). To improve the knockdown efficiency, several attempts were made. gRNA architecture has been proven to be essential for functioning of the CRISPR/Cas system (Liu et al., 2017). In eukaryotic transcription of gRNAs with the RNA Polymerase III promoter U6, the gRNA transcripts usually have a short stretch of four to eight Us at their 3′ end (Zhang C. et al., 2018). However, in the present prokaryotic transcription cases, intrinsic transcription terminator *T*_*rrnB*_ forms two consecutive self-annealing hairpin structures next to the second DR on the elongating transcript (Figure 4A). As a type IV-D CRISPR effector, CasRx first processes pre-gRNAs into mature gRNAs via a HEPN domain-independent mechanism (Zhang C. et al., 2018). We speculated that the hairpin structures of the prokaryotic gRNA terminator might disturb the binding of CasRx to pre-gRNA and processing of pre-gRNAs into mature gRNAs. Two strategies were applied to test our hypothesis. First, an additional sequence (approximately 100, 300, or 1,000 nt) was inserted between the second DR and the terminator in pre-gRNA. Second, the two 36-nt DRs with a 30-nt spacer in pre-gRNA were replaced by a 30-nt DR with a 22-nt spacer to mimic a mature gRNA (Figure 4A). Unfortunately, such modifications brought no positive effects to gene knockdown efficiency (Figures 4B,C). We further expanded the spacer length in mature gRNAs from 22 to 30 nt to enhance the potential binding between gRNA and target mRNA. However, this trial did not improve gene knockdown efficiency either (Supplementary Figure S3).

In some cases of dCas9- and dCas12a-mediated CRISPRi systems, the gene repression efficiency could be improved by a multiplex gRNAs strategy of making more than one gRNA toward the target gene simultaneously (Zhang J.L. et al., 2018). To test the effects of this strategy in this CRISPR/CasRx system, the most effective gRNA-3 was combined with gRNA-2 and gRNA-4, respectively. The resultant plasmids expressing a gRNA array were co-transformed with pCasRx-3 into *E. coli* harboring pGFP. The repression efficiencies observed were no higher than those obtained with an individual gRNA (Supplementary Figure S4). Considering the stoichiometry between CasRx-gRNA complex and the target mRNA, more weakly expressed genes may be repressed to a greater extent. We then constructed a pGFP-w plasmid using a weaker *P_*J*__23117_* promoter than the previously used *P_*J*__23105_*. By expressing CasRx and gRNA-3 in *E. coli* harboring pGFP-w, a knockdown efficiency of 56.1% was observed, which was slightly higher than the previously obtained ∼30% efficiency using the pGFP reporter system (Supplementary Figure S5).

## Discussion

Considering the importance of gene regulation in understanding and manipulating cellular metabolism, development of robust and efficient gene regulation techniques is drawing increasing attention. CRISPRi and RNAi that function at the transcription and translation stages, respectively, are well established and widely used in biological research and disease treatment (Laganà et al., 2014; Liu et al., 2018). With the advent of the type IV-D CRISPR effector (Cas13d), new programmable RNA-guided and RNA-targeting techniques are available, which have largely advanced the field of gene regulation (Wang F. et al., 2019). In this study, we demonstrate that the CRISPR/Cas13d system can be used for repressing gene expression in bacteria by knocking down target mRNA. However, the gene knockdown efficiency is far from satisfactory (30–50%) compared to those obtained in mammalian and plant cells (over 80%) (Konermann et al., 2018; Mahas et al., 2019). Although we optimized the gRNA architectures and combinations, the knockdown efficiency still hardly meets the requirements of practical application. Moreover, the CRISPR/Cas13d system seems highly inhibitory to microbial growth and metabolism.

The recently uncovered defense mechanism of type IV CRISPR systems against bacteriophages provides a possible explanation to the different performances of CRISPR/Cas13d in eukaryotic and prokaryotic cells (Jackson and Fineran, 2019; Meeske et al., 2019). Among the discovered CRISPR systems, type IV systems are intriguing because they are unique ones that cleave viral RNA rather than DNA. It was found that Cas13d induced cellular dormancy or even triggered cell death by degrading bacterial RNA, which aborted the infectious cycle and thus provided robust defense against bacteriophages (Meeske et al., 2019). Such collateral cleavage activity of the ternary Cas13d:gRNA:target RNA complex was verified *in vitro*, whereas it did not cause observable off-target transcriptome perturbation in mammalian and plant cells (Konermann et al., 2018; Mahas et al., 2019). Since these CRISPR systems are originated from bacterial adaptive immune systems, their distinct performances in eukaryotic and prokaryotic cells may be reasonable.

In conclusion, we developed an RNA knockdown technique based on the CRISPR/Cas13d system from *R. flavefaciens* XPD3002 (CRISPR/CasRx) and obtained moderate gene repression in *E. coli*. Considering that CRISPR/Cas13d directly acts on RNA and can process a CRISPR repeat array into multiple mature gRNAs without the involvement of additional RNases, this system holds promise for multiplex gene regulation in microbes. However, the collateral cleavage activity on bacterial RNA and relatively low knockdown efficiency need to be circumvented prior to practical applications. Recent successes in rational engineering and directed evolution of Cas proteins for increased activity, elevated targeting specificity, and released PAM requirements have set good examples for improving Cas13d (Kleinstiver et al., 2019; Miller et al., 2020).

## Data Availability Statement

The sequences for pCasRx-3, pgRNA-ccdB-1, and pgRNA-ccdB-2 were deposited into the GenBank database under the accession numbers MN934322, MN934323, and MN934324, respectively, at the National Center for Biotechnology Information. The full sequences for all the plasmids constructed in this study were uploaded as supplementary materials in GenBank format. All data and materials that support the findings of this study are available from the corresponding author on reasonable request.

## Author Contributions

YW, PZ, and JS conceived and initiated the project. YW and KZ designed the experiments. KZ, ZZ, JK, NG, and LF carried out the experiments. KZ, ZZ, YW, JC, and JL analyzed the data. YW, KZ, PZ, and JS wrote the manuscript. All authors read and approved the final manuscript.

## Conflict of Interest

The authors declare that the research was conducted in the absence of any commercial or financial relationships that could be construed as a potential conflict of interest.

## Footnotes

1 http://rna.tbi.univie.ac.at/
