# Supplementary material for: CRISPR/Cas13d-Mediated Microbial RNA Knockdown
Source: Front Bioeng Biotechnol. 2020 Jul 30;8:856. doi: 10.3389/fbioe.2020.00856 (PMC7406568; doi:10.3389/fbioe.2020.00856)
Supplement: Supplementary file 2 [file Data_Sheet_2.docx]

Supplementary Material

**TABLE S1.** Bacterial strains and plasmids used in this study.

| **Strain or plasmid** | **Description^a^** | **Reference or source** |
| --- | --- | --- |
| **Strain** |  |  |
| *E. coli* Trans1-T1 | General cloning host | TransGen |
| *E. coli* DB 3.1 | General cloning host for plasmids harboring *ccdB* gene | TransGen |
| *C. glutamicum* Δ*ldhA*::*gfp* | *C. glutamicum* ATCC 13032 derivative, *ldhA* replaced with a *gfp* expression cassette | Lab stock |
| **Plasmid** |  |  |
| pXMJ19 | *E. coli*-*C. glutamicum* shuttle vector, IPTG-inducible promoter *P_tac_*, *pUC* and *pBL1* replicons, Cm^R^ | ([Jakoby et al., 1999](#_ENREF_3)) |
| pEC-XK99E | *E. coli*-*C. glutamicum* shuttle vector, IPTG-inducible promoter *P_trc_*, *pUC* and *pGA1* replicons, Kan^R^ | ([Kirchner and Tauch, 2003](#_ENREF_4)) |
| pTrc99A | Expression vector, IPTG-inducible promoter *P_tac_*, *pUC* replicon, Amp^R^ | ([Amann et al., 1988](#_ENREF_1)) |
| pgRNA-*ccdB* | pEC-XK99E derivative, carrying gRNA::*ccdB* cassette driven by constitutive promoter *P_11F_* | ([Wang et al., 2018b](#_ENREF_7)) |
| pTRCmob-*egfp* | pTRCmob derivative, carrying *egfp* | ([Wang et al., 2018a](#_ENREF_6)) |
| pSB4K5-I52002 | BioBrick vector, *pSC101* replicon, Kan^R^ | ([Shetty et al., 2008](#_ENREF_5)) |
| pACYCDuet-1 | Expression vector, *p15A* replicon, Cm^R^ | Novagen |
| pZSA | Expression vector, tetracycline-inducible promoter *P_tet_*, *tetR*, *pSC101* replicon, Cm^R^ | Lab stock |
| pCasRx-1 | pXMJ19 derivative, carrying CasRx cassette driven by *P_tac_*, *pUC* and *pBL1* replicons | This study |
| pCasRx-2 | pCasRx-1 derivative, *pSC101* and *pBL1* replicons | This study |
| pCasRx-3 | pZSA derivative, carrying CasRx cassette driven by *P_tet_*, *pSC101* replicon | This study |
| pgRNA-*ccdB*-1 | pgRNA-*ccdB* derivative, carrying expression cassette for pre-gRNA of CasRx | This study |
| pgRNA-*ccdB*-2 | pgRNA-*ccdB* derivative, carrying expression cassette for mature gRNA of CasRx | This study |
| pgRNA-1 | pgRNA-*ccdB*-1 derivative, carrying *gfp*-targeting spacer gRNA-1 | This study |
| pgRNA-2 | pgRNA-*ccdB*-1 derivative, carrying *gfp*-targeting spacer gRNA-2 | This study |
| pgRNA-3 | pgRNA-*ccdB*-1 derivative, carrying *gfp*-targeting spacer gRNA-3 | This study |
| pgRNA-4 | pgRNA-*ccdB*-1 derivative, carrying *gfp*-targeting spacer gRNA-4 | This study |
| pgRNA-Control | pgRNA-*ccdB*-1 derivative, carrying non-targeting spacer (gRNA-Control) | This study |
| pgRNA-31 | pgRNA-*ccdB*-1 derivative, carrying *gfp*-targeting spacer and ~100-nt additional nucleotides (gRNA-31) | This study |
| pgRNA-32 | pgRNA-*ccdB*-1 derivative, carrying *gfp*-targeting spacer and ~300-nt additional nucleotides (gRNA-32) | This study |
| pgRNA-33 | pgRNA-*ccdB*-1 derivative, carrying *gfp*-targeting spacer and ~1,000-nt additional nucleotides (gRNA-33) | This study |
| pgRNA-m1 | pgRNA-*ccdB*-2 derivative, carrying *gfp*-targeting spacer gRNA-m1 | This study |
| pgRNA-m3 | pgRNA-*ccdB*-2 derivative, carrying *gfp*-targeting spacer gRNA-m3 | This study |
| pgRNA-mControl | pgRNA-*ccdB*-2 derivative, carrying non-targeting spacer gRNA-mControl | This study |
| pgRNA-m1N | pgRNA-*ccdB*-2 derivative, carrying *gfp*-targeting spacer gRNA-m1N | This study |
| pgRNA-m3N | pgRNA-*ccdB*-2 derivative, carrying *gfp*-targeting spacer gRNA-m3N | This study |
| pgRNA-mControlN | pgRNA-*ccdB*-2 derivative, carrying non-targeting spacer gRNA-mControlN | This study |
| pgRNA-3+2 | pgRNA-*ccdB*-1 derivative, carrying two *gfp*-targeting spacers gRNA-3 and gRNA-2 | This study |
| pgRNA-3+4 | pgRNA-*ccdB*-1 derivative, carrying two *gfp*-targeting spacers gRNA-3 and gRNA-4 | This study |
| pGFP | pTrc99A derivative, carrying *gfp* cassette driven by constitutive promoter *P_J23105_*, *p15A* replicon | This study |
| pGFP-w | pGFP derivative with *P_J23105_* replaced by *P_J23117_* for *gfp* expression | This study |

^a^Amp^R^, Kan^R^, and Cm^R^ represent resistance to ampicillin, kanamycin, and chloramphenicol, respectively.

**TABLE S2.** Primers used in this study.

| **Primer** | **Sequence (5’-3’)** | **Relevance** |
| --- | --- | --- |
| CasRx-F1 | TGGACAGGCTTTTTTCAGTAAGGAAGGCAG | pCasRx-2 |
| CasRx-R1 | acaatgtaacccagcaacgcggcctttttac |  |
| CasRx-F2 | ggtctgacagCGCAGAAAAAAAGGATCTCAAGAAG |  |
| CasRx-R2 | ACTGAAAAAAGCCTGTCCAAAGTAGCCGAG |  |
| pSC101-F | gcgttgctggGTTACATTGTCGATCTGTTCATGGTG |  |
| pSC101-R | tttttctgcgCTGTCAGACCAAGTTTACGAGCTC |  |
| CasRx-F3 | AGTGATAGAGATACTGAGCACAAAGGCACCCGATatga | pCasRx-3 |
| CasRx-R3 | cggatttgtcctactcagctatgaattacctgatactttcttc |  |
| pZSA-F | ctgagtaggacaaatccg |  |
| pZSA-R | GTGCTCAGTATCTCTATCACT |  |
| pgRNA-F | CCAGGCATCAAATAAAACGAAAG | pgRNA-*ccdB*-1  pgRNA-*ccdB*-2 |
| pgRNA-R | TGAATTACACTGTACCTGTTGCGTC |  |
| DR36-*ccdB*-F | aacaggtacagtgtaattcacaagtaaacccctaccaactggtcggggtttgaaacTGAGACCACGCGTGGATCC | pgRNA-*ccdB*-1 |
| DR36-*ccdB*-R | tcgttttatttgatgcctgggtttcaaaccccgaccagttggtaggggtttacttgTGAGACCTTATATTCCCCAGAACAT |  |
| DR30-*ccdB*-F | AACAGGTACAGTGTAATTCAAACCCCTACCAACTGGTCGGGGTTTGAAACTGAGACCACGCGTGGATCC | pgRNA-*ccdB*-2 |
| DR30-*ccdB*-R | TCGTTTTATTTGATGCCTGGTGAGACCTTATATTCCCCAGAACAT |  |
| gRNA-1-F | AAACTCCAGTGAAAAGTTCTTCTCCTTTACTCAT | pgRNA-1 |
| gRNA-1-R | CTTGATGAGTAAAGGAGAAGAACTTTTCACTGGA |  |
| gRNA-2-F | AAACAATTTAAGGGTAAGTTTTCCGTATGTTGCA | pgRNA-2 |
| gRNA-2-R | CTTGTGCAACATACGGAAAACTTACCCTTAAATT |  |
| gRNA-3-F | AAACACTTCAGCACGTGTCTTGTAGTTCCCGTCA | pgRNA-3 |
| gRNA-3-R | CTTGTGACGGGAACTACAAGACACGTGCTGAAGT |  |
| gRNA-4-F | AAACGTGGTCTCTCTTTTCGTTGGGATCTTTCGA | pgRNA-4 |
| gRNA-4-R | CTTGTCGAAAGATCCCAACGAAAAGAGAGACCAC |  |
| gRNA-Control-F | AAACcagggaatgaagaagctgtcgacgcgatta | pgRNA-Control |
| gRNA-Control-R | CTTGtaatcgcgtcgacagcttcttcattccctg |  |
| gRNA-3A-F | AAACACTTCAGCACGTGTCTTGTAGTTCCCGTCACAAGTAAACCCCTACCAACTGGTCGGGGTT | pgRNA-31  pgRNA-32  pgRNA-33 |
| gRNA-3A-R | TTCAAACCCCGACCAGTTGGTAGGGGTTTACTTGTGACGGGAACTACAAGACACGTGCTGAAGT |  |
| adn-F | CACACCAGGTCTCATGAAACAAACTCGCCGTTTATAGCAC | pgRNA-31  pgRNA-32  pgRNA-33 |
| adn-100-R | CACACCAGGTCTCACTTGAGCAGAAAGTCAAAAAATTCCAG | pgRNA-31 |
| adn-300-R | CACACCAGGTCTCACTTGATCATAGGCTGGAACACGGA | pgRNA-32 |
| adn-1000-R | CACACCAGGTCTCACTTGTTAAACCAGTTCGTTCGGG | pgRNA-33 |
| gRNA-m1-F | AAACaaagttcttctcctttactcat | pgRNA-m1 |
| gRNA-m1-R | CTGGatgagtaaaggagaagaacttt |  |
| gRNA-m3-F | AAACagcacgtgtcttgtagttcccg | pgRNA-m3 |
| gRNA-m3-R | CTGGcgggaactacaagacacgtgct |  |
| gRNA-mC-F | AAACcagggaatgaagaagctgtcga | pgRNA-mControl |
| gRNA-mC-R | CTGGtcgacagcttcttcattccctg |  |
| gRNA-1-F | AAACTCCAGTGAAAAGTTCTTCTCCTTTACTCAT | pgRNA-m1N |
| gRNA-m1N-R | CTGGATGAGTAAAGGAGAAGAACTTTTCACTGGA |  |
| gRNA-3-F | AAACACTTCAGCACGTGTCTTGTAGTTCCCGTCA | pgRNA-m3N |
| gRNA-m3N-R | CTGGTGACGGGAACTACAAGACACGTGCTGAAGT |  |
| gRNA-Control-F | AAACcagggaatgaagaagctgtcgacgcgatta | pgRNA-mControlN |
| gRNA-mCN-R | CTGGtaatcgcgtcgacagcttcttcattccctg |  |
| gRNA-32-1 | AAACACTTCAGCACGTGTCTTGTAGTTCCCGTCACA | pgRNA-3+2 |
| gRNA-32-2 | TACTTGTGACGGGAACTACAAGACACGTGCTGAAGT |  |
| gRNA-32-3 | AGTAAACCCCTACCAACTGGTCGGGGTTTGAAACAATTTAAGGGTAAGTTTTCCGTATGTTGCA |  |
| gRNA-32-4 | CTTGTGCAACATACGGAAAACTTACCCTTAAATTGTTTCAAACCCCGACCAGTTGGTAGGGGTT |  |
| gRNA-32-1 | AAACACTTCAGCACGTGTCTTGTAGTTCCCGTCACA | pgRNA-3+4 |
| gRNA-32-2 | TACTTGTGACGGGAACTACAAGACACGTGCTGAAGT |  |
| gRNA-34-3 | AGTAAACCCCTACCAACTGGTCGGGGTTTGAAACGTGGTCTCTCTTTTCGTTGGGATCTTTCGA |  |
| gRNA-34-4 | CTTGTCGAAAGATCCCAACGAAAAGAGAGACCACGTTTCAAACCCCGACCAGTTGGTAGGGGTT |  |
| pTrc99A-F1 | aagcttggctgttttggc | pGFP |
| pTrc99A-R1 | gcagaccaaaacgatctcaaatcttttctacggggtctga |  |
| pTrc99A-F2 | ggcggagcctatggaaatgttctttcctgcgttatcc |  |
| pTrc99A-R2 | CTAGTgctagcatagtacctaggactgagctagccgtaaatctagaggatccccgggta |  |
| GFP-F | aggtactatgctagcACTAGTGAAAGAGGAGAAATACTAGATGAGTAAAGGAGAAGAACTTTTCACTG |  |
| GFP-R | gccaaaacagccaagcttTTATTTGTATAGTTCATCCATG |  |
| p15A-F | ttgagatcgttttggtctgc |  |
| p15A-R | tttccataggctccgcc |  |
| J23117-F | TAGCTCAGTCCTAGGGATTGTGCTAGCACTAGTGAAAGAGGAG | pGFP-w |
| J23117-R | CAATCCCTAGGACTGAGCTAGCTGTCAATCTAGAGGATCCCCGG |  |
| pCasRx-2-vF | CTTAAAGGAGTTGAGAATGATCG | pCasRx-2 PCR verification |
| pCasRx-2-vR | GCTATGAATTACCTGATACTTTCTTC |  |
| pCasRx-3-vF | ATAGAGATTGACATCCCTATCA | pCasRx-3 PCR verification |
| pCasRx-3-vR | ctatgaattacctgatactttcttc |  |
| gRNA-1-F | AAACTCCAGTGAAAAGTTCTTCTCCTTTACTCAT | pgRNA PCR verification |
| gRNA-vR | CGCGCAAAATACTGCTCAGA |  |
| GFP-qF | CAACTAGCAGACCATTATCAAC | qPCR |
| GFP-qR | AACTCAAGAAGGACCATGTG |  |
| 16s-qF | CAGAATGCCACGGTGAATAC |  |
| 16s-qR | CGGTTACCTTGTTACGACTTC |  |

**TABLE S3.** Spacers used in this study.

| **gRNA** | **Spacer sequence of pre-gRNA (5’-3’)** | **Spacer sequence of mature gRNA (5’-3’)** |
| --- | --- | --- |
| gRNA-1 | TCCAGTGAAAAGTTCTTCTCCTTTACTCAT | TCCAGTGAAAAGTTCTTCTCCT |
| gRNA-2 | AATTTAAGGGTAAGTTTTCCGTATGTTGCA | AATTTAAGGGTAAGTTTTCCGT |
| gRNA-3 | ACTTCAGCACGTGTCTTGTAGTTCCCGTCA | ACTTCAGCACGTGTCTTGTAGT |
| gRNA-4 | GTGGTCTCTCTTTTCGTTGGGATCTTTCGA | GTGGTCTCTCTTTTCGTTGGGA |
| gRNA-Control | cagggaatgaagaagctgtcgacgcgatta | cagggaatgaagaagctgtcga |
| gRNA-m1 | aaagttcttctcctttactcat | aaagttcttctcctttactcat |
| gRNA-m3 | agcacgtgtcttgtagttcccg | agcacgtgtcttgtagttcccg |
| gRNA-mControl | cagggaatgaagaagctgtcga | cagggaatgaagaagctgtcga |
| gRNA-m1N | TCCAGTGAAAAGTTCTTCTCCTTTACTCAT | TCCAGTGAAAAGTTCTTCTCCTTTACTCAT |
| gRNA-m3N | ACTTCAGCACGTGTCTTGTAGTTCCCGTCA | ACTTCAGCACGTGTCTTGTAGTTCCCGTCA |
| gRNA-mControlN | cagggaatgaagaagctgtcgacgcgatta | cagggaatgaagaagctgtcgacgcgatta |

**TABLE S4.** Additional nucleotides of gRNA used in this study.

| **gRNA** | **Sequence of additional nucleotides (5’-3’)** |
| --- | --- |
| gRNA-31 | AAACTCGCCGTTTATAGCACAAAACAGTACGACAAGAAGTACCTGCAACAGGTGAACGAGTCCTTTGGCTTTGAGCTGGAATTTTTTGACTTTCTGCT |
| gRNA-32 | AAACTCGCCGTTTATAGCACAAAACAGTACGACAAGAAGTACCTGCAACAGGTGAACGAGTCCTTTGGCTTTGAGCTGGAATTTTTTGACTTTCTGCTGACGGAAAAAACCGCTAAAACTGCCAATGGCTGCGAAGCGGTATGTATTTTCGTAAACGATGACGGCAGCCGCCCGGTGCTGGAAGAGCTGAAAAAGCACGGCGTTAAATATATCGCCCTGCGCTGTGCCGGTTTCAATAACGTCGACCTTGACGCGGCAAAAGAACTGGGGCTGAAAGTAGTCCGTGTTCCAGCCTATGAT |
| gRNA-33 | AAACTCGCCGTTTATAGCACAAAACAGTACGACAAGAAGTACCTGCAACAGGTGAACGAGTCCTTTGGCTTTGAGCTGGAATTTTTTGACTTTCTGCTGACGGAAAAAACCGCTAAAACTGCCAATGGCTGCGAAGCGGTATGTATTTTCGTAAACGATGACGGCAGCCGCCCGGTGCTGGAAGAGCTGAAAAAGCACGGCGTTAAATATATCGCCCTGCGCTGTGCCGGTTTCAATAACGTCGACCTTGACGCGGCAAAAGAACTGGGGCTGAAAGTAGTCCGTGTTCCAGCCTATGATCCAGAGGCCGTTGCTGAACACGCCATCGGTATGATGATGACGCTGAACCGCCGTATTCACCGCGCGTATCAGCGTACCCGTGATGCTAACTTCTCTCTGGAAGGTCTGACCGGCTTTACTATGTATGGCAAAACGGCAGGCGTTATCGGTACCGGTAAAATCGGTGTGGCGATGCTGCGCATTCTGAAAGGTTTTGGTATGCGTCTGCTGGCGTTCGATCCGTATCCAAGTGCAGCGGCGCTGGAACTCGGTGTGGAGTATGTCGATCTGCCAACCCTGTTCTCTGAATCAGACGTTATCTCTCTGCACTGCCCGCTGACACCGGAAAACTATCATCTGTTGAACGAAGCCGCCTTCGAACAGATGAAAAATGGCGTGATGATCGTCAATACCAGTCGCGGTGCATTGATTGATTCTCAGGCAGCAATTGAAGCGCTGAAAAATCAGAAAATTGGTTCGTTGGGTATGGACGTGTATGAGAACGAACGCGATCTATTCTTTGAAGATAAATCCAACGACGTGATCCAGGATGACGTATTCCGTCGCCTGTCTGCCTGCCACAACGTGCTGTTTACCGGGCACCAGGCATTCCTGACAGCAGAAGCTCTGACCAGTATTTCTCAGACTACGCTGCAAAACTTAAGCAATCTGGAAAAAGGCGAAACCTGCCCGAACGAACTGGTTTAA |


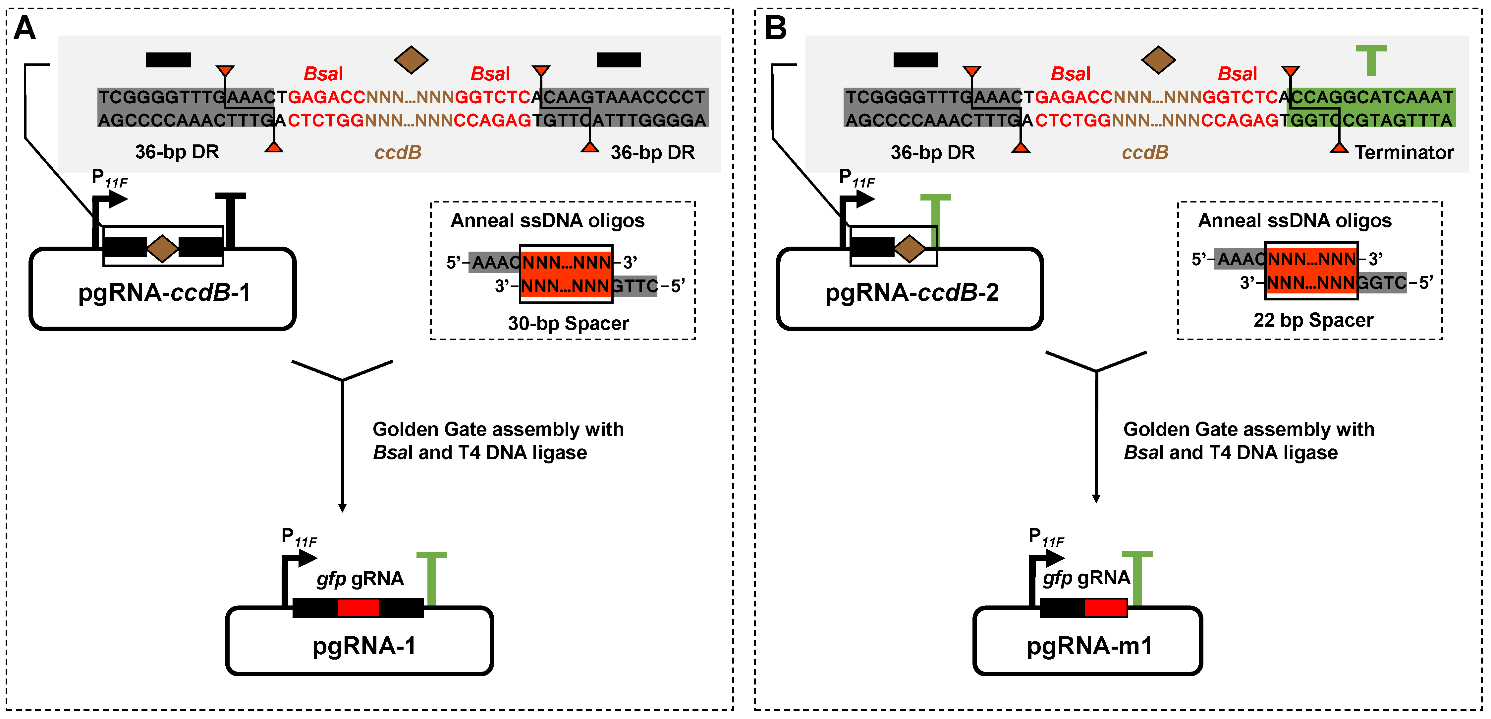


**FIGURE S1.** Construction procedure for gRNA expression plasmids. **(A)** pgRNA-*ccdB*-1 harbors a constitutive promoter (*P_11F_*), two 36-bp DRs (black rectangle), and a *ccdB* cassette (brown diamond) flanked by two *Bsa*I sites. As an example, the primer pair gRNA-1-F/gRNA-1-R (**Table S2**) was used to construct the 30-bp spacer brick by annealing of the two offset complementary ssDNA oligonucleotides. The 5’ ends of gRNA-1-F and gRNA-1-R were designed as sequences complementary to the sticky ends of *Bsa*I in plasmid pgRNA-*ccdB*-1. Golden Gate assembly was used to replace the *ccdB* cassette with the spacer brick, producing plasmid pgRNA-1. **(B)** pgRNA-*ccdB*-2 harbors a constitutive promoter (*P_11F_*), a 30-bp DR (black rectangle), and a *ccdB* cassette (brown diamond) flanked by two *Bsa*I sites. As an example, the primer pair gRNA-m1-F/gRNA-m1-R (**Table S2**) was used to construct the 22-bp spacer brick by annealing of the two offset complementary ssDNA oligonucleotides. The 5’ ends of gRNA-m1-F and gRNA-m1-R were designed as sequences complementary to the sticky ends of *Bsa*I in plasmid pgRNA-*ccdB*-2. Golden Gate assembly was used to replace the *ccdB* cassette with the spacer brick, producing plasmid pgRNA-m1.


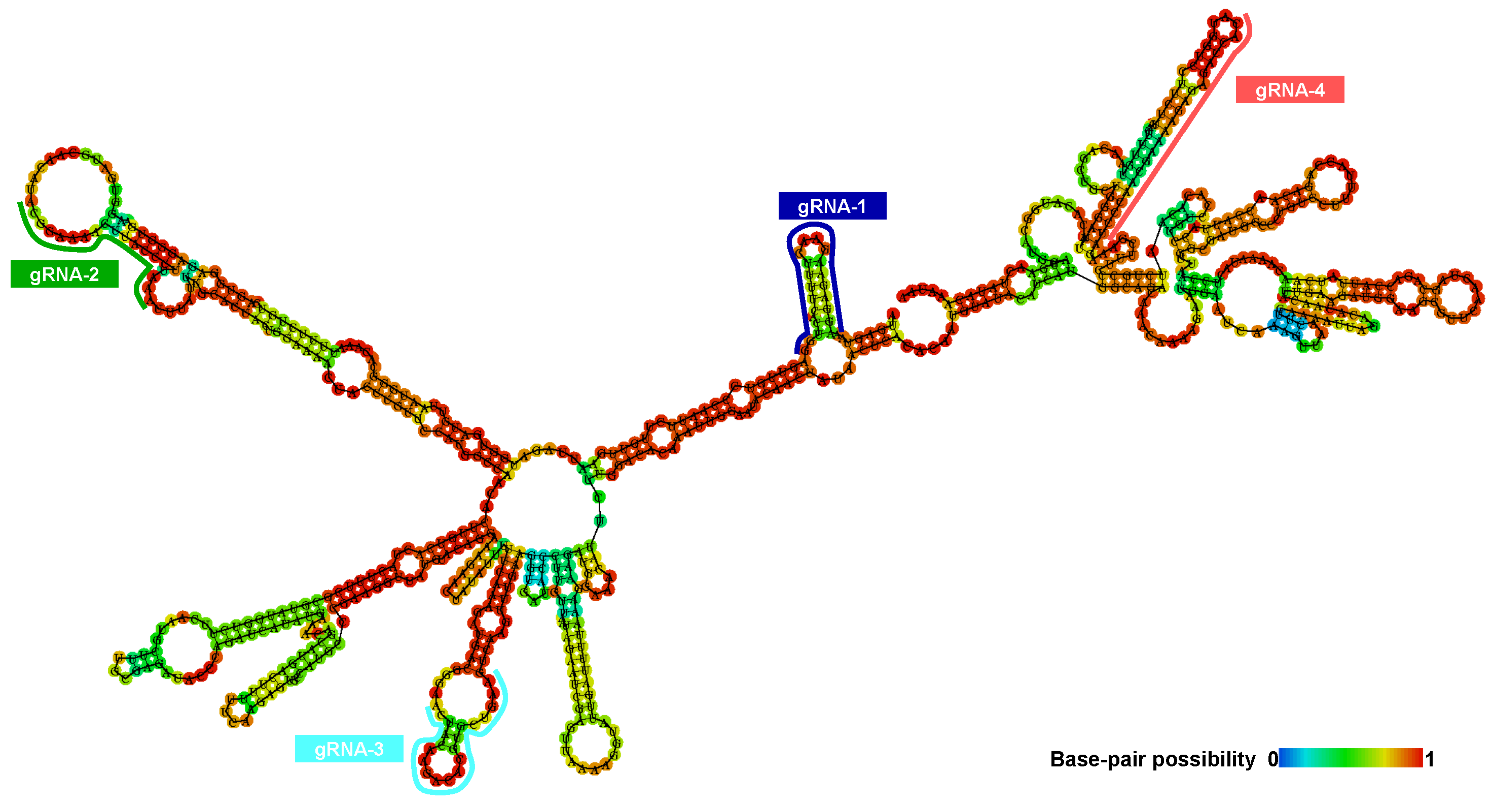
**FIGURE S2.** Predicted *gfp* mRNA structure and gRNA binding sites used in this study. Vienna RNA websuite (http://rna.tbi.univie.ac.at/) ([Gruber et al., 2008](#_ENREF_2)) was used to predict the secondary structure of *gfp* mRNA with the minimum free energy. The four gRNAs used in **Figure 1C** and **Figure 2** are highlighted.


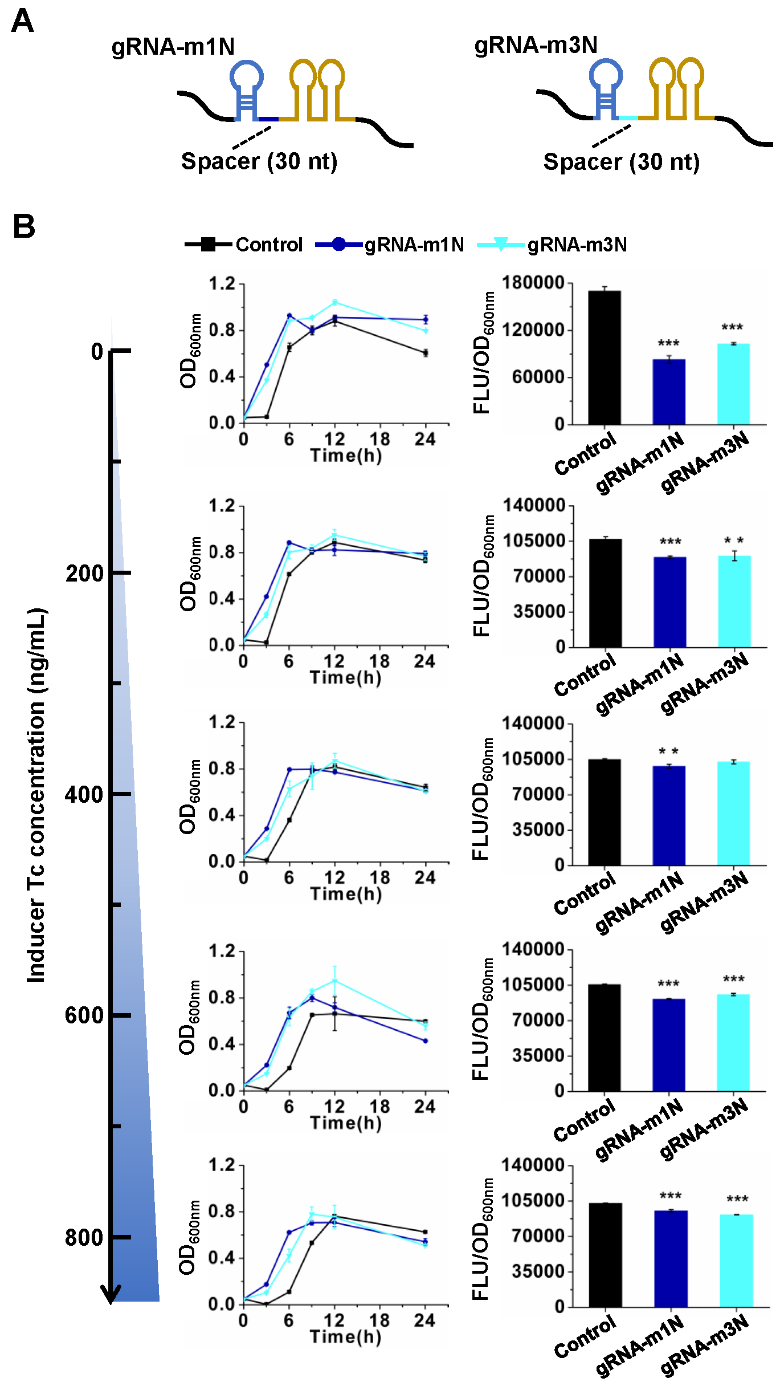


**FIGURE S3.** CRISPR/CasRx-mediated gene repression with mature gRNAs containing 30-nt spacers. **(A)** Architectures of mature gRNAs containing 30-nt spacers. gRNA-m1N and gRNA-m3N have the same spacers with gRNA-1 and gRNA-3, respectively, but different architectures. The spacer sequences are listed in **Table S3**. **(B)** Growth and GFP fluorescence with different gRNAs and inducer at different concentrations. pCasRx-3 and different gRNA expression plasmids were co-transformed into *E. coli* harboring pGFP for gene repression. Inducer was added at the beginning of cultivation to induce *casRx* expression. GFP fluorescence and OD_600nm_ were determined after 24 h cultivation. Error bars indicate standard deviations from three parallel experiments. All *t*-tests compare the GFP fluorescence per OD_600nm_ using *gfp*-targeting gRNAs against non-targeting gRNA control (***P*<0.01, ****P*<0.001).





**FIGURE S4.** CRISPR/CasRx-mediated gene repression with gRNA combinations. pCasRx-3 and plasmids expressing two gRNAs (gRNA-3 + gRNA-2 or gRNA-3 + gRNA-4) were co-transformed into *E. coli* harboring pGFP for gene repression. Inducer was added at the beginning of cultivation to induce *casRx* expression. GFP fluorescence and OD_600nm_ were determined after 24 h cultivation. Error bars indicate standard deviations from three parallel experiments. All *t*-tests compare the GFP fluorescence per OD_600nm_ using *gfp*-targeting gRNAs against non-targeting gRNA control (****P*<0.001).


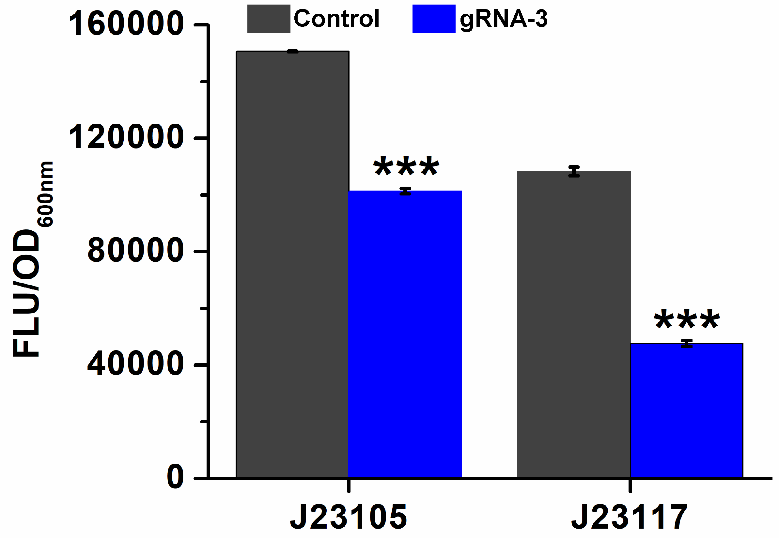


**FIGURE S5.** CRISPR/CasRx-mediated gene repression with a weakly expressed *gfp* reporter system. pCasRx-3 and pgRNA-3 were co-transformed into *E. coli* harboring pGFP (*gfp* controlled by *P_J23105_*) or pGFP-w (*gfp* controlled by *P_J23117_*) for gene repression. Inducer was added at the beginning of cultivation to induce *casRx* expression. GFP fluorescence and OD_600nm_ were determined after 24 h cultivation. Error bars indicate standard deviations from three parallel experiments. All *t*-tests compare the GFP fluorescence per OD_600nm_ using *gfp*-targeting gRNAs against non-targeting gRNA control (****P*<0.001).

# SUPPLEMENTARY REFERENCES

Amann, E., Ochs, B., and Abel, K.J. (1988). Tightly regulated tac promoter vectors useful for the expression of unfused and fused proteins in *Escherichia coli*. *Gene* 69(2), 301–315.

Gruber, A.R., Lorenz, R., Bernhart, S.H., Neubock, R., and Hofacker, I.L. (2008). The Vienna RNA websuite. *Nucleic Acids Res.* 36(Web Server issue), W70–W74. doi: 10.1093/nar/gkn188

Jakoby, M., Ngouoto-Nkili, C.-E., and Burkovski, A. (1999). Construction and application of new *Corynebacterium glutamicum* vectors. *Biotechnol. Tech.* 13(6), 437–441.

Kirchner, O., and Tauch, A. (2003). Tools for genetic engineering in the amino acid-producing bacterium *Corynebacterium glutamicum*. *J. Biotechnol.* 104(1-3), 287–299. doi: 10.1016/S0168-1656(03)00148-2

Shetty, R.P., Endy, D., and Knight, T.F., Jr. (2008). Engineering BioBrick vectors from BioBrick parts. *J. Biol. Eng.* 2, 5. doi: 10.1186/1754-1611-2-5

Wang, Y., Cao, G., Xu, D., Fan, L., Wu, X., Ni, X., et al. (2018a). A novel *Corynebacterium glutamicum* L-glutamate exporter. *Appl. Environ. Microbiol.* 84(6), e02691-17. doi: 10.1128/aem.02691-17

Wang, Y., Liu, Y., Liu, J., Guo, Y., Fan, L., Ni, X., et al. (2018b). MACBETH: multiplex automated *Corynebacterium glutamicum* base editing method. *Metab. Eng.* 47, 200–210. doi: 10.1016/j.ymben.2018.02.016
